# Temporal dynamics of linkage disequilibrium in two populations of bighorn sheep

**DOI:** 10.1002/ece3.1612

**Published:** 2015-07-23

**Authors:** Joshua M Miller, Jocelyn Poissant, René M Malenfant, John T Hogg, David W Coltman

**Affiliations:** 1 Department of Biological Sciences, University of AlbertaEdmonton, Alberta, Canada; 2 Centre for Ecology and Conservation, University of ExeterPenryn Campus, UK; 3 Montana Conservation Science Institute5200 Upper Miller Creek Road, Missoula, Montana, USA

**Keywords:** Admixture, D′, microsatellite, *Ovis canadensis*, relatedness, *χ*^′2^

## Abstract

Linkage disequilibrium (LD) is the nonrandom association of alleles at two markers. Patterns of LD have biological implications as well as practical ones when designing association studies or conservation programs aimed at identifying the genetic basis of fitness differences within and among populations. However, the temporal dynamics of LD in wild populations has received little empirical attention. In this study, we examined the overall extent of LD, the effect of sample size on the accuracy and precision of LD estimates, and the temporal dynamics of LD in two populations of bighorn sheep (*Ovis canadensis*) with different demographic histories. Using over 200 microsatellite loci, we assessed two metrics of multi-allelic LD, D′, and *χ*^′2^. We found that both populations exhibited high levels of LD, although the extent was much shorter in a native population than one that was founded via translocation, experienced a prolonged bottleneck post founding, followed by recent admixture. In addition, we observed significant variation in LD in relation to the sample size used, with small sample sizes leading to depressed estimates of the extent of LD but inflated estimates of background levels of LD. In contrast, there was not much variation in LD among yearly cross-sections within either population once sample size was accounted for. Lack of pronounced interannual variability suggests that researchers may not have to worry about interannual variation when estimating LD in a population and can instead focus on obtaining the largest sample size possible.

## Introduction

The goal of many conservation genetic, and now conservation genomic, studies is to identify genetic contributions to fitness differences within and among populations (Allendorf et al. 2010; Ouborg et al. 2010; Angeloni et al. 2012; Funk et al. 2012; McMahon et al. 2014), possibly narrowing genetic effects down to causative loci. Frequently used analytical approaches include outlier analyses (Namroud et al. 2008; Nosil et al. 2012), detection of inbreeding through heterozygosity fitness correlations (HFCs; Grueber et al. 2011; Szulkin et al. 2010; Miller and Coltman 2014), and identifying adaptive genetic variation or evolutionary potential (Funk et al. 2012; Hansen et al. 2012; Harrisson et al. 2014). These findings can help guide management actions aimed at preserving genetic diversity and population persistence (Shafer et al. 2015).

Key to the success of such endeavors is having the correct number of genetic markers to accurately associate phenotypes with genotypes, or to characterize population parameters (e.g., inbreeding). With the advent of high-throughput sequencing technology (Metzker 2010), continuing decline in sequencing costs (Glenn 2011), and new methods for simultaneously generating novel loci and genotypes (Baird et al. 2008; Hohenlohe et al. 2010; Elshire et al. 2011), large panels of markers can now be developed for most species. However, one key parameter that has received relatively little attention in the conservation community is linkage disequilibrium (LD). LD is the nonrandom association of alleles between two loci, and patterns of LD have both biological implications, for example, by determining the effect of selection on neutral sites linked to those under selection, as well as practical implications when designing studies. Specifically, long stretches of LD allow for detection of associations between genotypes and phenotypes with fewer markers, but impede fine mapping of associations to specific genes or variants. Thus, LD determines the number of markers needed to obtain adequate coverage in a genome-wide association study (GWAS; Stram 2004), HFC study (Miller et al. 2011), or outlier analyses (Luikart et al. 2003), as well as the precision one may hope to achieve for fine mapping an association that has been found (Carlson et al. 2004; Stram 2004). It is therefore important to quantify LD in wild populations and understand what causes it to vary.

Linkage disequilibrium has been quantified in a handful of studies in the wild (e.g., Backström et al. 2006; Slate and Pemberton 2007; Li and Merilä 2010; Miller et al. 2014), and other aspects of LD are beginning to be examined, such as differences between populations (Balakrishnan and Edwards 2009; Li and Merilä 2011; Yang et al. 2014). Theoretical and empirical examinations have shown that the magnitude and extent of LD are influenced by many factors including admixture, inbreeding, recombination rate, and genetic diversity (Lewontin 1964, 1995; Weir 1979; Brookes 1999; Pritchard and Przeworski 2001; Ardlie et al. 2002; Mueller 2004; Slatkin 2008; Gray et al. 2009). However, LD is not a species or population specific constant; like heterozygosity and heritability, it is a characteristic of a population measured at one point of time. The temporal dynamics of linkage disequilibrium have received little empirical attention, especially at short time scales. Cross-generational LD was examined in commercial chicken (*Gallus gallus*) lines, where it was found to be stable (Heifetz et al. 2005). In contrast, Slate and Pemberton (2007) found considerable interannual variability in background LD (LD among nonsyntenic markers) in a wild population of red deer (*Cervus elaphus*) following an admixture event. A better understanding of the temporal dynamics of LD in wild populations is needed to determine whether LD-based predictions of the optimal number of loci required for an association or outlier study can be extrapolated across time periods.

**Figure 1:**
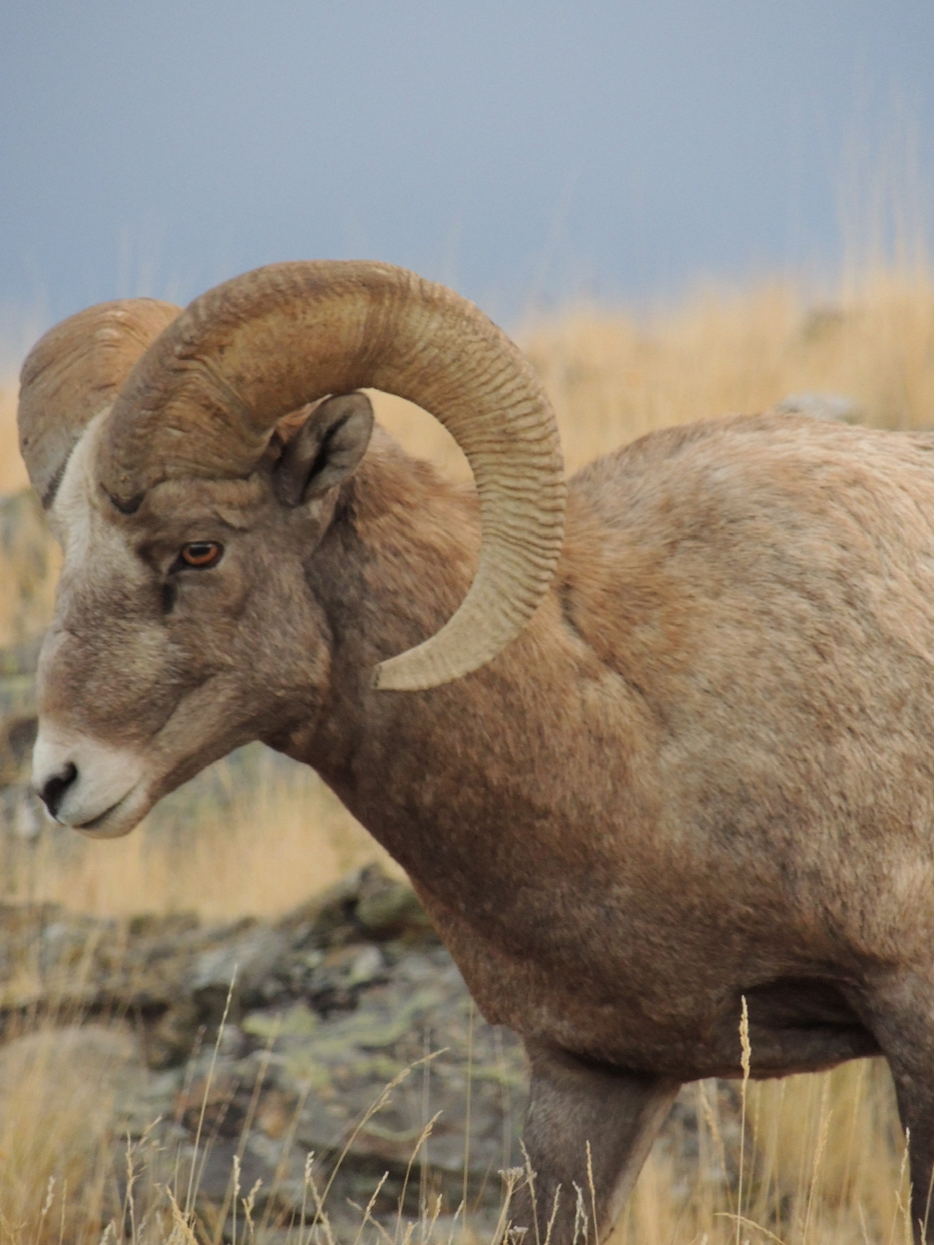
A bighorn ram from National Bison Range MT, USA.

In this study, we examined the temporal patterns of LD using over 200 microsatellite markers genotyped in two populations of bighorn sheep (*Ovis canadensis*; Fig. 1) with different demographic histories: one a native population; the other founded via translocation, which then experienced a prolonged bottleneck post founding, followed by recent admixture. These two populations are both the subject of long-term studies and thus represent a unique opportunity to examine year-by-year patterns of LD in wild populations. We also examine the effect of sample size on the accuracy and precision of LD estimates. This consideration is important for researchers wishing to conduct a pilot LD study using as few individuals as possible, which will then dictate the marker density used in future population-wide analyses. Similarly, our results can inform cases where obtaining large sample sizes may be impractical, such as for populations or species of conservation concern. We expected the level of LD in the native population to be lower and more stable than in the translocated population, which we predicted will have higher LD and show more interannual variability due to the bottleneck and admixture.

## Methods

### Study populations

We examined patterns of linkage disequilibrium in two populations: Ram Mountain (Alberta, Canada; RM) and at National Bison Range (Montana, USA; NBR). Both populations are the subjects of long-term studies where individuals are followed throughout their lives, and each population has an associated pedigree where relationships among individuals are determined through field observations as well as genetic analyses (Poissant et al. 2010).

Ram Mountain is a native population in which individual-based monitoring began in 1972 with genetic sampling starting in 1988 (Jorgenson et al. 1997, 1993; Coltman et al. 2003). Between 1972 and 1989, census size increased from ∼100 sheep to ∼220. However, since the 1990s, the population has been in a steady decline, currently numbering ∼60 individuals, due to low recruitment (Jorgenson et al. 1993) and cougar (*Puma concolor*) predation (Festa-Bianchet et al. 2006). In 2004 and 2007, a total of 17 sheep from a neighboring population were introduced to RM (Rioux-Paquette et al. 2010). None of these individuals or their progeny were included in our analyses. Thus, we do not expect admixture to play a significant role in temporal patterns of LD observed in RM. In contrast, NBR was founded in 1922 via translocation of 12 individuals from Banff National Park (Alberta, Canada). Individual monitoring started in 1979, with genetic sampling starting in 1988. Beginning in 1985, NBR experienced a “genetic rescue” via intentional translocation of individuals (*N* = 15) from neighboring populations (Hogg et al. 2006; Miller et al. 2012). Prior to the introduction, census size and growth rate had been steadily declining (average census size of 48 sheep between 1922 and 1985); however, following the supplementation, there has been an increase both in census size (142 sheep at end-of-year 2012) and in genetic diversity (Hogg et al. 2006; Miller et al. 2012).

### Marker genotyping and selection

Microsatellite markers used in this study were selected from those previously genotyped for the construction of a genetic linkage map (Poissant et al. 2009). Primer information and PCR conditions for the markers can be found in Poissant et al. (2010, 2009) and references therein. For each population, we included all mapped autosomal markers that had no more than 15% missing genotypes. In total, 208 and 210 markers met these criteria in the RM and NBR populations, respectively; 192 of which were typed in both populations. Intermarker distances, measured in centimorgans (cM), were taken from the combined map (Poissant et al. 2009) and therefore are assumed to be equal between populations. In both populations, the final dataset contained an average (±SD) of 8 ± 4 markers per chromosome. Average intermarker distance was 15.58 cM ± 10.04 cM in RM and 15.68 cM ± 10.34 cM in NBR. As with markers, we excluded any individual with more than 15% missing genotypes. In total, 276 and 216 individuals were included for RM and NBR, respectively.

### Quantifying linkage disequilibrium

We considered two measures of LD: D′, and *χ*^′2^. D′ is a commonly used measure of LD in studies with multi-allelic markers (Slate and Pemberton 2007; Lipkin et al. 2009; Li and Merilä 2010, 2011), as it is easy to calculate and standardized by the maximal allele frequencies in the population (Hedrick 1987; Zapata 2000; McRae et al. 2002). An alternative metric, *χ*^′2^ (Zhao et al. 2002), is thought to more accurately reflect associations between genetic markers and quantitative trait loci (QTL). As has been commonly found (e.g., Slate and Pemberton 2007; Li and Merilä 2011), *χ*′^2^ yielded lower values than D′ but similar qualitative patterns (see Results) and we present both metrics to facilitate comparison to previous studies.

In population studies, LD should ideally be estimated using unrelated individuals. However, because we were most interested in obtaining a snapshot of LD which would be obtained by sampling individuals over only a few years in the absence of a priori knowledge about (cryptic) relatedness, we estimated LD with a population-based approach assuming that individuals were unrelated. For this, we used the gap package version 1.1 (Zhao 2005) in R (R Development Core Team 2005). Specifically, D′ and *χ*^′2^ were calculated using the LDkl function based on estimates of haplotype frequencies obtained for each pair of markers using the genecounting function (Zhao et al. 2002).

Patterns of LD in each population were summarized using two metrics. First, we calculated background LD, which we calculated as the average LD between pairs of nonsyntenic markers (*n* = 20,557). Second, we calculated half-length (Reich et al. 2001), or rate of decay: the distance (in cM) over which LD declines by half. To calculate half-lengths, we used the exponential decay function described in De La Vega et al. (2005) using syntenic marker pairs separated by less than 50 cM (*n* = 436 and 431 in RM and NBR, respectively). Specifically, LD was modeled as a function of intermarker distances (*d*) using the formula


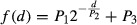


where *P*_1_ is a scalar, *P*_2_ is the rate of decay (or half-length), and *P*_3_ is the background, or asymptotic, level of LD. In all models, *P*_3_ was set to LD measured using nonsyntenic markers, and *P*_1_ was set to *b* − *P*_3_, where *b* is the intercept. When working with physical distance, intercepts (LD at 0 base pairs) should arguably be set to 1. However, this is not necessarily so when working with recombination fractions (cM) as markers in complete linkage (0 cM) are not necessarily expected to be in perfect LD because of historical or undetected recombination. For example, in our datasets, average D′ ± 1 standard deviation (SD) for the 4 marker pairs that were completely linked (0 cM) was 0.65 ± 0.15 in RM and 0.76 ± 0.08 in NBR. For *χ*^′2^, the average was 0.36 ± 0.18 in RM and 0.39 ± 0.14 in NBR. Therefore, we estimated intercepts from the data rather than constraining them to 1. As *P*_3_ was already known, we only solved for *P*_1_ (i.e., *b*) and *P*_2_ using the nls function in the stats package in R. Because estimates of background LD (and possibly half-lengths) are strongly influenced by sample size (Slate and Pemberton 2007), values from the two populations could not be compared directly. To characterize the influence of sample size on LD measures and allow comparison of LD between populations, we estimated background LD and half-lengths in each population by subsampling equal number of individuals (10, 25, 50, 75, 100, 125, 150, 175, 200) 1000 times.

### Factors influencing the extent of LD

We used linear models to examine what factors influenced the overall patterns of LD among syntenic markers (those on the same chromosome and separated by less than 50 cM). Specifically, we tested whether LD (D′ and *χ*^′2^ values based on all samples pooled) was influenced by intermarker distance (cM, log transformed), chromosome (a factor with 26 levels), and the average heterozygosity of the two markers. Average heterozygosity has been found to be positively correlated with LD (McRae et al. 2002; Slate and Pemberton 2007), as reduced heterozygosity can mask crossover events. Chromosome was included because previous research has found significant interchromosomal differences in the extent of LD (e.g., De La Vega et al. 2005; Li and Merilä 2010, 2011). The number of individuals genotyped at a marker pair is known to influence LD (McRae et al. 2002; Slate and Pemberton 2007), but this was not considered here, as there was little variation in the amount of missing data among markers (average 2% ± 3% SD in both populations).

Model simplification was conducted via an information theoretic approach (Burnham and Anderson 2002; Grueber et al. 2008) using MuMIn version 1.7.2 (Bartoń 2009) in R. Specifically, we used the dredge() function and assessed model differences with ΔAICc values. We restricted analysis to models less than 2 AICc from the top model.

### Temporal variation in LD

In order to study temporal variation in LD, we calculated background LD and half-length for yearly cross-sections. As expected, in both populations, we observed strong relationships between yearly sample size and LD measures (see Results). However, additional correlations between sample size and year, especially in NBR, prevented us from satisfactorily separating the influence of sample size and year on LD measures (data not shown). To get around this difficulty, we decided to estimate LD at different time points using a fixed number of individuals. Specifically, for each year in both RM and NBR, we estimated background LD and half-lengths using subsamples of 20 individuals 500 times. Doing so provided results that were unbiased by sample size and therefore comparable across time and populations. However, it must be stressed that absolute values generated in that way are still biased by sample size and therefore are not discussed in absolute terms.

To investigate whether temporal patterns could be due to variation in relatedness, we also estimated temporal variation in mean relatedness using the same approach. Relatedness was calculated based either on marker genotypes or directly from the pedigree of each population. For marker-based relatedness, we used Coancestry v1.0.0.1 (Wang 2011). Allele frequencies for each population were calculated from the full dataset, and we accounted for inbreeding using 500 bootstraps and 100 reference individuals. In each yearly cross-section, we recorded pairwise estimates of two relatedness metrics: one moment estimator, Queller and Goodnight (1989), and one measure which accounts for inbreeding in the sample, Trio ML (Wang 2011). For pedigree-based relatedness, we extracted pairwise relatedness estimates for all sampled individuals using the R package Pedantics version 1.04 (Morrissey and Wilson 2010). However, we present results considering only Queller and Goodnight given that all the metrics considered were highly correlated, and the majority of wild populations will not have a pedigree available.

## Results

**Figure 2:**
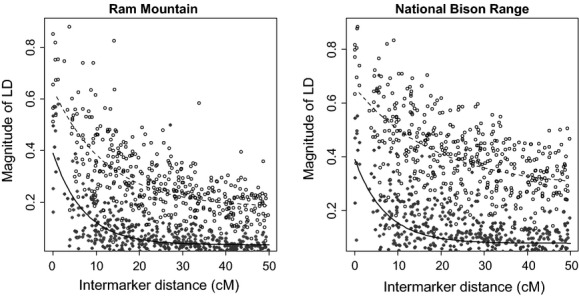
Plots of D′ (open circles) and *χ*^′2^ (closed circles) as a function of intermarker distance (cM) in RM and NBR. Lines are from an exponential decay function (see text for details).

In general, D′ and *χ*^′2^ showed similar patterns, but the magnitude of LD estimates was lower with *χ*^′2^ (Fig. 2). When considering all individuals simultaneously, background LD ± 1 SD measured using D′ and *χ*^′2^ were 0.18 ± 0.07 and 0.04 ± 0.03 in RM, and 0.29 ± 0.08 and 0.08 ± 0.04 in NBR. LD decreased with increasing intermarker distance in both populations. In RM, the half-length, or rate of decay, ± 1 standard error (SE) across all samples pooled was 8.63 ± 0.56 cM for D′ and 4.90 ± 0.29 cM for *χ*^′2^. In NBR, the half-length was 12.80 ± 1.01 cM for D′ and 8.79 ± 0.70 cM for *χ*^′2^.

**Figure 3:**
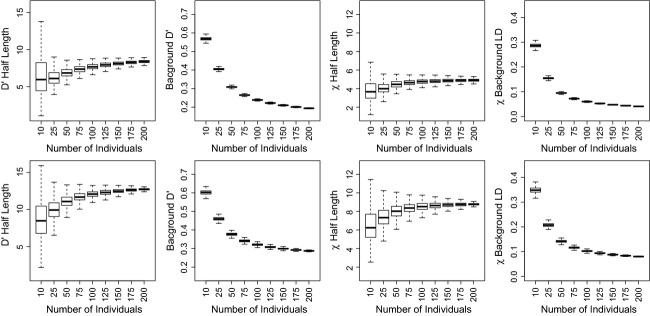
Box plots of effect of sample size on estimates of background LD and half-length (cM) in RM (top row) and NBR (bottom row). Errors bars show SD from 1000 bootstraps.

Estimating background LD by subsetting different number of individuals confirmed an expected strong negative relationship between background LD and sample size (Fig. 3). The effect of sample size on background LD estimates was similar for both LD measures, being very pronounced at small sample size and becoming marginal once sample size reached 100 individuals. Our analysis also highlighted a positive relationship between half-length and sample size. However, overall, the relationship between sample size and half-length was much less pronounced than for background LD, and in particular appeared to plateau at smaller sample sizes. Differences in LD between NBR and RM using equal number of individuals (Fig. 3) demonstrated that background LD and half-lengths were larger in NBR compared to RM. Overall, the magnitude of background LD was ∼30% larger, and half-lengths were ∼3 cM longer in NBR than in RM.

The factors influencing LD among syntenic markers were largely consistent between populations and LD metrics (Table 1). As mentioned above, LD decreases with increasing intermarker distance. In contrast, LD increases with increasing average heterozygosity between the markers. Lastly, we found that LD did not differ among chromosomes, except for *χ*^′2^ in RM where the top model containing chromosome as a factor was 12.20 AICc away from the model that did not contain chromosome.

**Table 1:** Top two models exploring factors that explain differences in LD between syntenic markers

	Intercept	Chr^1^	Distance^2^	Hz^3^	df	logLik	AICc	delta	weight
RM									
D′	0.28		−0.18	0.05	4	333.76	−659.4	0.00	0.99
	0.29	+	−0.17	0.05	29	356.37	−650.5	8.98	0.01
*χ*^′2^	0.08	+	−0.09	0.05	29	566.13	−1070	0.00	1.00
	0.08		−0.09	0.04	4	532.93	−1057.8	12.20	0.00
NBR									
D′	0.41		−0.15	0.08	4	333.82	−659.5	0.00	1.00
	0.44	+	−0.15	0.08	29	351.55	−640.9	18.67	0.00
*χ*^′2^	0.15		−0.11	0.04	4	450.15	−892.2	0.00	0.75
	0.16	+	−0.11	0.06	29	476.11	−890.0	2.22	0.25

For each term, the effect sizes have been standardized on 2 SD (Gelman 2008).

1 Chromosome, fit as a factor with 26 levels.

2 Intermarker distance (measured in centimorgans).

3 Mean heterozygosity of the two markers contributing to each estimate of marker–marker LD.

**Figure 4:**
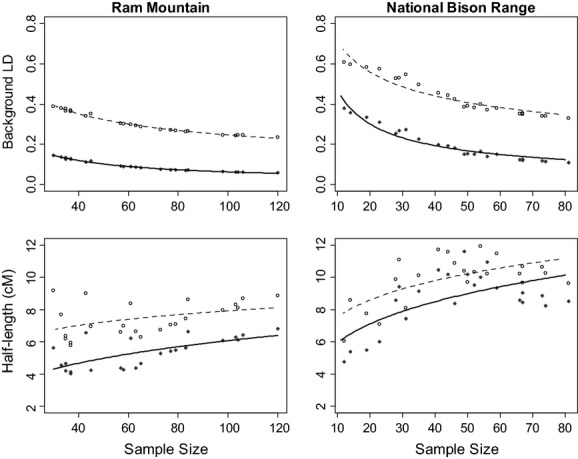
Plots of D′ (open circles) and *χ*^′2^ (closed circles) as a function of sample size in RM and NBR. The lines are from a power function *f*(*x*) = *cx*^*r*^ where *c* and *r* were determined using the R nls function.

Interannual variation in background LD was principally explained by differences in sample size (Fig. 4 top row). In linear models where sample size was modeled using a power function, r^2^ values between sample size and LD were very large in both populations for both metrics (all *r*^2^ > 0.90, all *P* < 0.001). In contrast, variation in half-length was much less influenced by sample size (Fig. 4 bottom row), which is consistent with observations made when subsetting population samples (i.e., Fig. 3). For NBR, *r*^2^ was 0.43 for both LD metrics (both *P* < 0.001), while in RM, *r*^2^ was 0.15 for D′ (*P* = 0.06) and 0.45 (*P* < 0.001) for *χ*^′2^.

**Figure 5:**
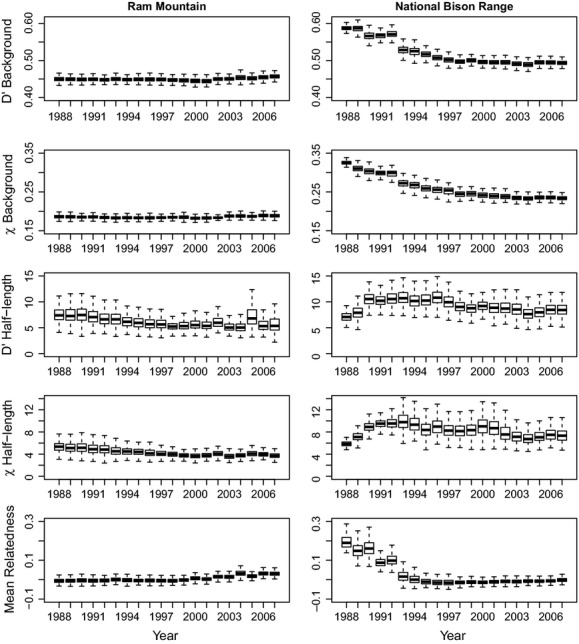
Box plots of temporal variation in background LD, half-lengths, and relatedness in RM and NBR. Yearly estimates were obtained by subsampling 20 individuals 500 times. Errors bars show SD from 500 bootstraps.

When temporal patterns were analyzed using a fixed number of individuals, both LD metrics (D′ and *χ*^′2^) showed similar patterns in background LD and half-lengths within populations (Fig. 5). There was little evidence for large interannual changes in LD through time in RM, although there appeared to be a downward trend in half-lengths. In contrast, LD in NBR appears more dynamic. Background LD decreased rapidly between 1988 and 1998, followed by a slower rate of decline between 1999 and 2007, while half-lengths in 1988 and 1989 appeared to be much lower than in subsequent years. However, it is important to note that in 1988 and 1989, sample sizes were small (close to 20 individuals), so subsampling 500 sets of individuals may not provide more accurate estimates as the same individuals are included again and again. This could lead to an upward bias in background LD and downward bias in half-length estimates. Yearly values of mean relatedness were significantly correlated with all measures of background LD, but only with one measure of half-length: *χ*^′2^ in RM (Table 2).

**Table 2:** Pearson's product-moment correlations between yearly estimates of relatedness and measures of LD

	Correlation	*t*-value	*P*-value^1^
RM			
Background D′	0.75	4.84	<0.001
Background *χ*^′2^	0.72	4.34	<0.001
D′ Half-length	−0.38	−1.74	0.10
*χ*^′2^ Half-length	−0.58	−3.03	0.01
NBR			
Background D′	0.94	11.95	<0.001
Background *χ*^′2^	0.93	11.06	<0.001
D′ Half-length	−0.09	−0.40	0.69
*χ*^′2^ Half-length	−0.16	−0.70	0.49

1 Values based on 18 degrees of freedom.

## Discussion

In this study, we conducted an analysis of the extent and temporal patterns of LD in two populations of bighorn sheep with different population histories. Using sets of over 200 microsatellite loci, we found that both populations exhibited extensive LD, although the extent was much shorter in a native population (RM) than in one which experienced recent admixture via a genetic rescue after years of inbreeding (NBR). Bootstrap analysis also highlighted that using a small number of individuals will generally result in imprecise and biased LD estimates. Analyses of LD in yearly cross-sections showed that there was little appreciable temporal variation the extent of LD in either population after differences in sample size were accounted for.

### Factors influencing the extent of LD

Both populations exhibited high levels of LD compared to estimates for other wild populations (Backström et al. 2006; Laurie et al. 2007; Balakrishnan and Edwards 2009; Gray et al. 2009; Stapley et al. 2010), although see Li and Merilä (2010, 2011) who found the half-length of LD extended for 1–5 cM in Siberian jay (*Perisoreus infaustus*) populations. High levels of LD are likely a function of bighorn sheep life history characteristics. Specifically, bighorn sheep have a polygynous mating system where the majority of offspring are sired by a minority of males (Hogg and Forbes 1997; Coltman et al. 2002). In addition, sheep tend to be highly philopatric (Rioux-Paquette et al. 2010). Together, these factors are likely to lead to nonrandom mating and therefore extend the levels of LD throughout the genome (Terwilliger et al. 1998; Slatkin 2008). Estimates of LD in NBR were larger than those in RM, which was expected given the unique population history: Descendants of NBR founders were shown to have low overall genetic diversity after years of inbreeding, and then experienced admixture with translocated individuals from neighboring herds (Hogg et al. 2006; Miller et al. 2012); both of these processes should lead to nonrandom association of alleles among loci (Nei and Li 1973; Terwilliger et al. 1998; Slatkin 2008). Such interpopulation differences have been seen in studies of both wild and domestic organisms (McKay et al. 2007; Meadows et al. 2008; Balakrishnan and Edwards 2009; Li and Merilä 2011; Yang et al. 2014) and were similarly linked to differences in population history. In addition to the species-specific factors and demographic events listed above, it is likely that genetic drift has also led to the high levels of LD in these populations. Genetic drift is expected to generate LD simply through random sampling of alleles from generation to generation (Ohta 1982; Terwilliger et al. 1998; Slatkin 2008), a process that is exacerbated in small populations.

As expected, LD was found to decay with increasing intermarker distance. In addition, we found that LD increased with average heterozygosity. Other research has suggested lower average heterozygosity should increase LD, as runs of homozygosity would mask crossover events (Song et al. 2009; Li and Merilä 2010). However, our work is consistent with previous work in domestic sheep (*Ovis aries*), which showed a positive correlation between D′ and heterozygosity (McRae et al. 2002). The authors attributed this association to rare alleles at highly heterozygous markers increasing estimates of LD. There may also be an effect of admixture in the case of NBR, in that the introductions increased levels of heterozygosity and also the levels of LD.

The different measures of half-length were similarly variable within the two populations: D′ = 8.63 ± 0.56 cM and *χ*^′2^ = 4.90 ± 0.29 cM for RM versus D′ = 12.80 ± 1.01 cM and *χ*^′2^ = 8.79 ± 0.70 cM for NBR. Although striking, differences in the magnitude of LD as measured by D′ and *χ*^′2^ have been seen in previous studies that considered both measures (Slate and Pemberton 2007; Li and Merilä 2010, 2011). Note that the estimate of *χ*^′2^ half-length in RM was similar to a previous estimate based on single nucleotide polymorphisms (SNPs), ∼4.6 mega base pairs (Miller et al. 2014), assuming that 1 cM is approximately 1 mega base pair (Dumont and Payseur 2008).

### Temporal patterns

For the most part, we did not observe large changes in LD estimated between years in either population. Surprisingly, average relatedness in each yearly cross-section was found to be associated with all measures of background LD, but only one measure of half-length in one population.

In RM, the general decrease in half-lengths may be the result of population level processes associated with a decrease in population size experienced over the past two decades, such as inbreeding or genetic drift (Nei and Li 1973; Ohta 1982; Terwilliger et al. 1998; Slatkin 2008).

The fact that temporal patterns of LD in NBR were also relatively stable ran contrary to our expectations. We had expected the temporal patterns of LD to reflect the complex demographic history of the NBR: historical inbreeding followed by three rounds of introductions of unrelated sheep. As discussed above, the small values for half-lengths in 1988 and 1989 may simply be a result of small sample sizes in these years. Without the influence of sample size, we would have expected extensive LD in these years as a result of complete segregation between the “introduced” and “founder” genomes. Although not as dynamic as we expected, background LD did show some variation over time. Here, background LD starts at a relatively high level, likely a result of the first introduction in 1985. However, rather than simply declining after this maximal value, a plateau is evident from 1990 to 1992 followed by a substantial decline in 1993. Previous empirical and simulation studies have shown that following an introduction, there is a spike in the level of LD due to the complete segregation between the “introduced” and “founder” genomes. LD then decreases over time as the introduced genome is backcrossed with the founder genome (Nei and Li 1973; Pfaff et al. 2001; Slate and Pemberton 2007). Interestingly, the plateau loosely corresponds with a subsequent introduction in 1990. The fact that additional introductions in 1993 and 1994 (*N* = 6 and 3 individuals respectively) did not cause abrupt increases in the extent of LD may be because all of these individuals were taken from neighboring Montana herds that were founded using the same population those used as the initial 1985 translocation to NBR. Therefore, it is possible that a spike in LD was prevented due to an already high representation of “introduced genomes” in NBR.

### Implications for association studies

The power to detect associations between genotypes and phenotypes is a complex interplay between the effect size of the QTL and the level of LD in the population. A QTL will be detected with fewer markers if there is substantial LD, although long tracks of LD will impede fine mapping of associations. Differences between populations (Balakrishnan and Edwards 2009; Li and Merilä 2011; this study; Yang et al. 2014) indicate that there is no canonical level of LD. Rather, it is a dynamic measure that depends on population history. While this is to be expected, it may not explicitly be accounted for in the design of association studies.

The long stretches of LD seen in both RM and NBR indicate that fewer markers will be needed for association analyses within these two populations, and likely other bighorn sheep populations with similar demographic histories (Johnson et al. 2011; Olson et al. 2013). However, determining the exact number will depend not only on the population, but also the metric used to estimate the extent of LD. Given the apparent ubiquity of differences in magnitude of LD as measured by D′ and *χ*^′2^ (Li and Merilä 2010, 2011; Slate and Pemberton 2007; this study), this situation is likely to be faced by other researchers. Determining the optimal marker density based on a specific metric will involve weighing the possibility of missing an association due to insufficient coverage against the cost of developing and typing additional markers which may prove to be redundant.

Another consideration is the sample size needed in pilot studies where LD is measured to determine the optimal number of markers for downstream studies. Our bootstrap analysis showed that small sample sizes will under estimate half-lengths, which may lead researchers to use more markers than are truly necessary. Therefore, for the most accurate estimate of LD, a relatively large number of samples should be used, for the populations we consider here ∼100 individuals. In the case of small populations, this may mean combining multiple cohorts.

One factor we do not explicitly consider here is the choice of marker. SNPs are rapidly emerging as the marker of choice for association analyses (Slate et al. 2010; Helyar et al. 2011; Santure et al. 2013), while all of our results are based on a genome-wide set of microsatellite loci. However, we do not see this as a problem given that we are most interested in the dynamics of LD over time and examining what factors lead to those patterns. The factors we investigated here (admixture, sample sizes, and relatedness among individuals) are likely to drive patterns of LD among SNPs as well as microsatellite loci. However, we acknowledge that the specific estimates of the extent of LD in each population may change when considering SNPs and physical distances relative to microsatellites and cM. Nevertheless, it is interesting to note that we found a similar estimate of half-length for RM when measured with microsatellites using the *χ*^′2^ measure and SNPs (Miller et al. 2014).

## Conclusion

Both populations of bighorn sheep examined in this study exhibited extensive LD, the magnitude of which reflected their unique histories. Our results add to the growing body of evidence that there is not a canonical level of LD for a species. Rather, LD varies between populations, but perhaps not within them over short time scales. By necessity, pilot studies or studies of endangered species or small populations will often use relatively few samples. Our results indicate that such studies will tend to underestimate LD and consequently overestimate the number of markers needed for association analyses. However, our observation of the relative stability of LD over time means that researchers may not have to be concerned about large interannual variation in LD and can instead focus on collecting enough samples for a less biased estimate of LD.

## Data Accessibility

Microsatellite genotypes have been deposited on Dryad http://dx.doi.org/10.5061/dryad.n2cj3.

## Supporting Information

**Table S1.** List of marker names and genomic locations.
